# Can artificial intelligence improve medicine’s uncomfortable relationship with Maths?

**DOI:** 10.1038/s41746-024-01168-8

**Published:** 2024-06-22

**Authors:** Alexandra Valetopoulou, Simon Williams, Hani J. Marcus

**Affiliations:** 1 Wellcome/EPSRC Centre for Interventional and Surgical Sciences, University College London, London, UK; 2 Department of Neurosurgery, National Hospital for Neurology and Neurosurgery, London, UK

**Keywords:** Medical research, Epidemiology

In 1978, Casscells et al. posed a medical statistics question to healthcare professionals that highlighted medicine’s uncomfortable relationship with statistics^1^. They were asked the following question:

“*If a test to detect a disease whose prevalence is 1/1000 has a false positive rate of 5%, what is the chance that a person found to have a positive result actually has the disease, assuming that you know nothing about the person’s symptoms or signs*?”.

The results showed that only a minority provided the correct answer, with most clinicians overestimating the positive predictive value (PPV)^1^. The study was replicated by Manrai et al. 36 years later, yielding similar results, highlighting that medical statistics continue to challenge healthcare professionals, irrespective of grade, despite advancements in medical education^2^.

ChatGPT is an advanced natural language processing generative artificial intelligence (AI) model trained on large-scale data to produce human like responses^3^. We aim to replicate these two studies with the addition of AI assistance, comparing respondent accuracy and confidence with and without AI assistance.

Twenty attendings, 20 interns/residents, and 20 final-year medical students participated. Initially, the correct answer was given by 10 of 60 participants (17%) (Table 1, Fig. 1). Similar results were obtained in the study by Manrai et al. (14 of 61 correct answers, 23%), and in the study by Casscells et al. (11 of 60 correct answers, 18%). In all three studies the most common answer was 95%, given by 34 of 60 (57%) of respondents in this study, 27 of 61 (44%) in the Manrai et al. study, and 27 of 60 (45%) in the Casscells et al. study.

**Table 1:** Proportion of correct answers and respondent confidence in their answer with and without AI-assistance

	Without AI-assistance	AI-assistance	*p* value
Proportion of correct answers (%)	10/60 (17%)	30/36 (83%)	<0.001
Respondent confidence (median [IQR])	2 (1–3)	4 (3–4)	<0.001

**Fig. 1:**
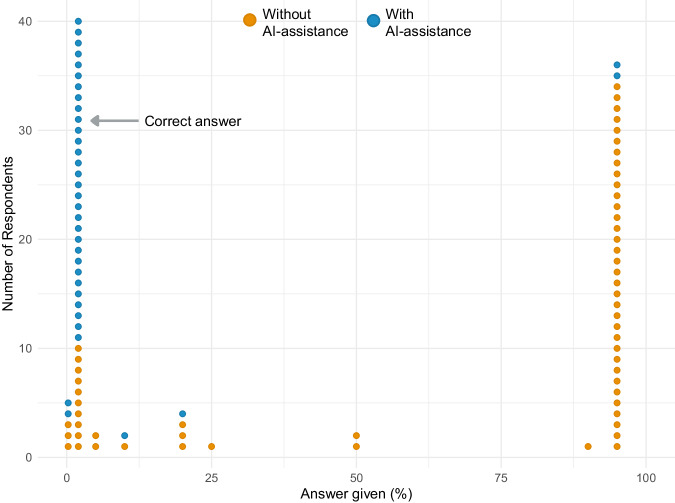
Distribution of answers with and without AI-assistance. The plot demonstrates the distribution of answers provided with AI-assistance (blue) and without AI-assistance (orange).

Thirty-six of 60 (60%) participants modified their answer after viewing the AI response, with 30 of 36 (83%) providing the correct answer when given AI-assistance (Table 1, Fig. 1). There was a significant increase in the proportion of correct answers with AI-assistance (*p* < 0.001).

The median confidence ranking was 2 - ‘slightly confident’ (IQR 1–3) when participants answered the question with no assistance and 4 - ‘fairly confident’ (IQR 3–4) when given AI assistance (Table 1). There was a significant increase in confidence with AI-assistance (*p* < 0.001).

Despite a 45-year gap between the original study and our study, most healthcare professionals remain unable to correctly calculate the PPV. However, we observed a significant improvement in accuracy and confidence in answers when respondents were given AI-assistance.

With increasing development and implementation of clinician decision support (CDS) algorithms, clinicians require sound probabilistic reasoning skills to interpret CDS outputs and integrate them into clinical decision making^4^. Given healthcare professionals remain challenged by medical statistics, modern approaches to teach and interpret probabilities are needed.

In this study, participants’ confidence in their answer increased with AI-assistance. However, it is important to consider whether the tool led to improved knowledge and understanding, or if participants simply trusted the AI generated response. Exploring the mechanisms which facilitate statistical learning and understanding using generative AI is crucial before tools are implemented within medical education and clinical practice.

Teaching medical students how to best use generative AI, with a focus on leveraging practical, real-world scenarios^5^, may enrich understanding. This may equip future healthcare professionals with the skills to apply generative AI in their clinical practice – encouraging data-driven decision-making.

## Methods

### Survey

We conducted a survey of attendings, interns/residents, and final-year medical students from a range of medical and surgical specialties, at a tertiary center in the UK using convenience sampling. Participants initially answered the question without assistance. They were then shown the ChatGPT response and asked whether they would modify their initial answer. At each stage respondents ranked confidence in their answer on a 1–5 scale (1 - not confident at all; 5 - very confident). We calculated the correct answer to be 1.96%, and to ensure consistency with the previous studies we also considered ‘2%’ as correct^2^. The question stem was inputted to ChatGPT-3.5, which generated a step-by-step response and the correct answer (Supplementary Note 1).

### Analysis

We used the chi-squared test to compare for difference in the proportion of correct answers, and the Mann–Whitney U test to compare for difference in confidence.

### Reporting summary

Further information on research design is available in the Nature Research Reporting Summary linked to this article.

### Supplementary information

Supplementary Information

Reporting summary

## Data Availability

The data that support the findings of this study are available from the corresponding author upon request.

## Code availability

We used the pre-existing ChatGPT-3.5 software. The prompt for the ChatGPT-3.5 output used in this study is described in the supplementary note 1.

## References

[1] Casscells W, Schoenberger A, Graboys TB (1978). Interpretation by physicians of clinical laboratory results. N. Engl. J. Med..

[2] Manrai AK, Bhatia G, Strymish J, Kohane IS, Jain SH (2014). Medicine’s uncomfortable relationship with math: calculating positive predictive value. JAMA Intern. Med..

[3] OpenAI. ChatGPT (June 2023 Version) [Large language model].

[4] Goodman KE, Rodman AM, Morgan DJ (2023). Preparing physicians for the clinical algorithm era. N. Engl. J. Med..

[5] Miles S, Price GM, Swift L, Shepstone L, Leinster SJ (2010). Statistics teaching in medical school: opinions of practising doctors. BMC Med. Educ..

