# Supplementary material for: Can artificial intelligence improve medicine’s uncomfortable relationship with Maths?
Source: NPJ Digit Med. 2024 Jun 22;7:166. doi: 10.1038/s41746-024-01168-8 (PMC11193742; doi:10.1038/s41746-024-01168-8)
Supplement: Supplementary file 1 — Supplementary Information [file 41746_2024_1168_MOESM1_ESM.pdf]

Supplementary Note 1: Chat-GPT response to the statistical question

"To calculate the chance that a person found to have a positive result actually has the disease, we can use Bayes' theorem. Bayes' theorem relates conditional probabilities and can be expressed as follows:
P(A|B) = (P(B|A) * P(A)) / P(B)

Where: P(A|B) is the probability of event A occurring given that event B has occurred.
P(B|A) is the probability of event B occurring given that event A has occurred.
P(A) is the probability of event A occurring.
P(B) is the probability of event B occurring.

In this case, let's define the events as follows:
A: Person has the disease.
B: Person tests positive.

Given the information provided:
P(A) = 1/1000 (prevalence of the disease)
P(B|A) = 1 (probability of testing positive given that the person has the disease)
P(B|¬A) = 0.05 (probability of testing positive given that the person does not have the disease)
P(¬A) = 1 - P(A) = 1 - 1/1000 (probability of not having the disease)

To calculate P(B), we need to consider the total probability of testing positive:
P(B) = P(B|A) * P(A) + P(B|¬A) * P(¬A)
= 1 * (1/1000) + 0.05 * (999/1000)
= 0.001 + 0.04995
≈ 0.05095

Now we can calculate P(A|B) using Bayes' theorem:
P(A|B) = (P(B|A) * P(A)) / P(B)
= (1 * (1/1000)) / 0.05095
≈ 0.0196

Therefore, the chance that a person found to have a positive result actually has the disease is approximately 0.0196 or 1.96%."
